# *GanCtrl:* a generative AI approach to derive study-aligned synthetic controls for reducing concurrent control animal use

**DOI:** 10.1093/toxsci/kfag099

**Published:** 2026-08-06

**Authors:** Mansi Chandra, Xi Chen, Ting Li, Weida Tong

**Affiliations:** National Center for Toxicological Research, Food and Drug Administration, Jefferson, AR 72079, United States; University of Arkansas at Little Rock and University of Arkansas for Medical Sciences Joint Bioinformatics Program, Little Rock, AR 72204, United States; National Center for Toxicological Research, Food and Drug Administration, Jefferson, AR 72079, United States; National Center for Toxicological Research, Food and Drug Administration, Jefferson, AR 72079, United States; National Center for Toxicological Research, Food and Drug Administration, Jefferson, AR 72079, United States

**Keywords:** 3Rs, concurrent control groups (CCGs), virtual control groups (VCGs), generative AI, generative adversarial networks (GANs), toxicology

## Abstract

Minimizing the use of animals as concurrent controls in in vivo studies directly supports the 3Rs principles of Replacement, Reduction, and Refinement. Current virtual control group (VCG) approaches primarily rely on historical control data, but their utility may be limited by cross-study variability from differences in study design, laboratory practices, and data heterogeneity. We propose GanCtrl, a generative AI approach to infer study-specific control data directly from time-matched treatment data. By generating synthetic controls analogous to concurrent controls, GanCtrl aims to mitigate biological, temporal, and technical biases inherent in VCG approaches based on historical control data. GanCtrl was applied to rat repeat-dose toxicity studies to simulate 38 clinical pathology endpoints under control conditions using corresponding treatment data. Synthetic controls closely approximated real concurrent controls, with differences smaller than intra- and inter-laboratory baseline variation and comparable to biological replicate variability, while also preserving the typical magnitude and distribution of control responses across studies. Importantly, synthetic controls enabled detection of drug-induced clinical pathology signals and maintained biologically relevant endpoint relationships, such as ALT–AST. For practical utility, toxicity assessments using GanCtrl-derived synthetic controls were compared with those using real concurrent controls and benchmarked against VCGs constructed from single-laboratory or combined multi-laboratory historical data. Although both approaches performed comparably in the single-laboratory setting, GanCtrl outperformed VCGs when data from multiple laboratories were combined. These findings suggest that GanCtrl offers a potential approach for generating VCGs that may reduce the use of concurrent control animals and advance the 3Rs.

Animal studies have long been central to toxicology and provide essential evidence for the safety and risk assessment of chemicals and drugs (Mangipudy et al. 2014; Mukherjee et al. 2022). However, the regulatory landscape is rapidly evolving toward reduced reliance on animal use. Specifically, there is increasing emphasis on New Approach Methodologies (NAMs) as alternatives to animal models for toxicity assessment (Kaplan et al. 2024). These efforts align with the 3Rs (Replacement, Reduction, and Refinement) principle and regulatory initiatives such as the FDA’s *Roadmap to Reducing Animal Use in Nonclinical Safety Studies* (Russell et al. 1959; Carratt et al. 2025; de Zafra and Carosino 2025). Replacement efforts include ex vivo models (e.g. the bovine corneal opacity and permeability test), in vitro systems (e.g. liver organ-on-chip), in silico approaches (e.g. read-across and quantitative structure–activity relationship models), and in chemico assays (e.g. the direct peptide reactivity assay) (Caloni et al. 2022; Zushin et al. 2023; Ouedraogo et al. 2025). Yet, despite substantial progress, it is challenging for NAMs to fully recapitulate the biological complexity, systemic interactions, or longitudinal dynamics captured in whole-animal studies (Shrimali et al. 2025). In parallel, meaningful advances have been made in refining animal studies through optimized study designs and shortened dosing regimens (Ackley et al. 2023; Chien et al. 2023). However, because refinement strategies primarily improve how animal studies are conducted rather than replace core study components, they may have limited impact on total animal numbers when statistically powered concurrent control groups are still required for study validity. At the same time, efforts are increasingly focused on reduction strategies that decrease animal use while maintaining scientific reliability.

For example, initiatives such as BioCelerate’s Toxicology and Background Control Data Sharing initiative enable precompetitive sharing of nonclinical toxicology and background control data to improve compound progression decisions and reduce animal use through optimized study design (Houser and Mangipudy 2019). Noticeably, the EU-funded IHI VICT3R consortium (Developing and Implementing Virtual Control Groups to Reduce Animal Use in Toxicology Research) assembled historical control datasets and developed methods for replacing concurrent control groups with modeled virtual control groups (VCGs) in standard safety studies (Steger-Hartmann et al. 2025). It has been anticipated that such approaches could substantially reduce the number of animals required, potentially by ∼25% in standard toxicology designs, without compromising statistical validity (Mecklenburg et al. 2023; Golden et al. 2024; Li et al. 2024a).

Currently, VCGs have gained prominence in the community to minimize animal use based on historical control datasets (Steger-Hartmann et al. 2020; Steger-Hartmann et al. 2025). Despite its theoretical appeal and potential utility, current VCG methodologies face certain challenges. A major limitation lies in the heterogeneity of historical control datasets, driven by genetic, environmental, and procedural variability across studies and laboratories (Greim et al. 2003). Variations in analytical platforms and clinical chemistry methods further complicate cross-study harmonization and temporal alignment (Crabbe et al. 1999; Steger-Hartmann et al. 2020). Moreover, the statistical foundations of current VCG models face challenges in capturing nonlinear, multivariate biological interactions and the physiological interdependence among measurements (Grevot et al. 2023). These limitations motivate exploration of more adaptive, biologically grounded computational paradigms with modern AI methodologies.

Rapid advances in generative AI (GenAI) have enabled the synthesis of complex, high-dimensional biological data by learning underlying data distributions using methods such as Variational Autoencoders (VAEs) and Generative Adversarial Networks (GANs). This capability has been exemplified by AnimalGAN and ToxGAN, which generated synthetic rat clinical pathology and transcriptomic profiles closely aligned with experimentally observed compound-specific effects (Chen et al. 2022, 2023). In addition, GenAI is able to translate the data across biological context as demonstrated with studies for cross-organ transcriptomic translation (Li et al. 2023, 2024b) and in vitro to in vivo extrapolation (Chandra et al. 2025). We hypothesize that time-matched control information can be inferred from the corresponding treatment data using the similar GenAI-based translational methodology.

In this proof-of-concept study, we developed GanCtrl, a GenAI approach using a GAN-like architecture to infer clinical pathology profiles of controls from their time-matched treatment conditions with data from the rat in vivo repeat-dose studies conducted at 4 time points (3, 7, 14, and 28 days) (Igarashi et al. 2015). We assessed the statistical performance by comparing “synthetic control” against the data from real controls. We specifically examined how the toxicity assessments based on synthetic controls agreed with those based on real concurrent controls across 14 clinically relevant clinical pathology endpoints and benchmarked against VCG methods. Our results demonstrated that GanCtrl can generate practically useful synthetic controls that approximate real controls with potential as an alternative approach for constructing VCGs in reducing the use of concurrent control animals to advance the 3Rs principle.

## Materials and methods

### Dataset description

Clinical pathology data for both treatment groups and their time-matched controls were obtained from in vivo repeated-dose studies in male Sprague-Dawley rats that included treatment durations of 3, 7, 14, and 28 days, with up to 5 biological replicates per condition (Igarashi et al. 2015). A total of 38 clinical pathology measurements were used in the modeling process, including 17 hematologic parameters and 21 clinical chemistry parameters. Among the clinical chemistry measures, 7 are liver-associated (ALT, AST, ALP, TBIL, DBIL, LDH, and GTP) and 7 are kidney-related (blood urea nitrogen [BUN], creatinine [CRE], Na, K, Cl, Ca, and IP) (Chen et al. 2023). Full names for these liver- and kidney-related measurements are provided in Table S1. GanCtrl was focused on inferring the clinical pathology profiles of controls from their time-matched highest dose group in treatment data. Specifically, the dataset included 2,737 vehicle-treated control samples and 2,597 treated samples from 138 unique compounds. Rat body weight measurements were also retrieved and incorporated as a physiological covariate to support context-aware model conditioning. Chemical structure was represented using 1,613 Mordred-derived 2-dimensional (2D) molecular descriptors for each compound (Moriwaki et al. 2018). All data were obtained from https://dbarchive.biosciencedbc.jp/en/open-tggates/download.html.

### Pair construction and train-test split

To enable treatment-to-control translation, each treated sample (3/7/14/28 day) was paired with all vehicle control replicates from the same compound and time point to form treatment–control pairs, ensuring proper temporal alignment of physiological and biochemical responses. This process yielded a total of 12,935 treatment–control pairs. The dataset was then randomly split by compound to prevent data leakage, allocating 80% (110 compounds) for model training and 20% (28 compounds) for independent testing. This resulted in 10,369 training pairs and 2,566 test pairs for model development and evaluation, respectively.

### GanCtrl model development, training, and selection

#### GanCtrl architecture

We developed GanCtrl as a Conditional Variational Autoencoder–Generative Adversarial Network (CVAE–GAN) comprising 3 coordinated components, an encoder, a biologically informed decoder, and a discriminator, trained jointly to translate treatment profiles into time-matched control data.

##### Encoder

The encoder received as input a concatenated vector containing treatment data across 38 clinical pathology measurements, together with conditioning variables describing experimental context and compound molecular descriptors. All quantitative variables were normalized to a 0 to 1 range using a MinMax scaler prior to GanCtrl model training. The conditioning vectors included binary encodings for the source (treatment) and target (control) domains consisting of categorical indicators for sacrifice time point and biological replicate identity, a continuous body-weight covariate and a 1-hot cluster label used as an input condition for prediction. Cluster labels were defined by *k*-means clustering of training control samples; during training, treatment samples inherited the cluster labels of their concurrent control samples, whereas during inference, test samples were assigned labels by mapping them to the nearest cluster centroid learned from the training data. To incorporate chemical information, the encoder additionally received each compound’s Mordred-derived 2D molecular descriptor vector, which was transformed via a learned dense embedding network into a lower-dimensional representation and concatenated with the other inputs. The encoder outputs a mean and log-variance defining a latent Gaussian distribution, from which a stochastic latent vector was sampled via the reparameterization trick.

##### Decoder

The decoder was designed as an attention-aware, biologically informed network that reconstructs control profiles over the 38 clinical pathology measurements. Inputs are the latent vector from encoder concatenated with the treatment data across 38 clinical pathology measurements, and the same contextual labels used by the encoder. An attention-based feature-mixing layer operates under a predefined biological neighbor mask that restricts attention to curated measurement neighborhoods (e.g. hepatic injury enzymes ALT, AST, LDH, and renal function measurements BUN, CRE), limiting information flow to biologically plausible neighborhoods.

The mixed representation was passed through a shared backbone consisting of fully connected layers with batch normalization, LeakyReLU activations, and dropout. From this backbone, a set of sub-decoders—each corresponding to a control cluster—learned cluster-specific feature transformations. The final prediction was obtained by combining the backbone-derived base output with the cluster-specific residual, capturing both global structure and cluster-dependent effects. The underlying mathematical details are provided in the Supplementary Methods.

##### Discriminator

The discriminator was implemented as a lightweight conditional classifier to distinguish synthetic controls from real controls under matched experimental contexts. It received either a synthetic control or real control feature vector together with contextual encodings for time point and biological replicate identity. The network consisted of 3 fully connected layers with ReLU activations and dropout regularization, followed by a sigmoid output neuron estimating the probability that each input corresponded to a real control sample. Trained using binary cross-entropy loss with mild label smoothing, the discriminator provided adversarial feedback to the decoder, encouraging the generation of physiologically realistic, control-like feature profiles.

##### Training objective

The GanCtrl model was optimized with a multi-component objective balancing reconstruction accuracy, biological realism, and physiological plausibility. The total loss was:


$$L_{t\mathrm{otal}}=L_{\mathrm{NLL}}++\beta_{KL}L_{KL}+L_{\mathrm{adv}}+L_{\mathrm{range}}+L_{\mathrm{bio}-\mathrm{corr}}+L_{\mathrm{mean}-\mathrm{match}}$$

Each component serves a distinct role. We include a variation reconstruction term ($L_{\mathrm{NLL}}+{\beta_{KL}L}_{KL})$ comprising a negative log-likelihood term and Kullback–Leibler divergence to fit control-equivalent predictions while regularizing the latent space and stabilizing sample-variance learning; adversarial term ($L_{\mathrm{adv}})$ to guide synthetic controls to be indistinguishable from real controls, thereby enhancing realism and distributional alignment; a physiological range constraint term ($L_{\mathrm{range}})$ to keep synthetic controls within empirically observed control bounds; biological correlation preservation term ($L_{\mathrm{bio}-\mathrm{corr}})$ to maintain co-regulated measurement relationships (e.g. ALT–AST–LDH, BUN–CRE); and mean-matching regularization term ($L_{\mathrm{mean}-\mathrm{match}})$ that encourages predicted control means to converge toward empirical control means. Further mathematical formulations for each loss are provided in the Supplementary Methods.

##### Model selection

We selected model checkpoints using a composite score that combined statistical fidelity metrics and biological coherence summaries into a single trajectory over training epochs. For each organ, we *z*-normalized the evaluation metrics across checkpoints and combined them into a weighted score curve. We then selected a checkpoint from the first stable region of consistently low scores to avoid transient dips. An elbow/plateau heuristic was used to confirm training stabilization, and the final checkpoint was the step with the lowest adjusted score, with neighboring steps inspected as a sanity check. A detailed specification of the composite score is provided in the Supplementary Methods.

### Model evaluation

#### GanCtrl versus real control data

Quantitative agreement between synthetic controls and real control profiles was evaluated using cosine similarity and root mean squared error (RMSE). Cosine similarity measured the alignment between the 2 profiles, whereas RMSE captured the magnitude of average deviations, with lower values indicating higher accuracy. Cosine similarity and RMSE were computed between each synthetic control and its corresponding real control profile to quantify how closely GanCtrl replicated the real-control structure. In addition, we applied Welch’s 2-sample *t*-test to assess statistical significance of differences between groups (Welch 1947).

We also evaluated whether substituting synthetic controls for real controls preserves biological inferences, focusing on literature-anchored hepatotoxic and nephrotoxic patterns. For each treatment, we tested the hypothesis $H_{1}:\mu_{\mathrm{treatment}}>\mu_{\mathrm{control}}\;$using a one-sided Welch *t*-test, applied twice: First comparing treatment vs. real controls, and then comparing treatment vs. synthetic controls. Across the 14 hepatotoxicity- and nephrotoxicity-related measurements, *P*-values were adjusted using the Benjamini–Hochberg procedure (Benjamini and Hochberg 1995) to obtain FDR-adjusted *q*-values. A measurement was considered elevated if q≤ 0.05, and the mean difference was non-negative. These measurement-level elevation calls were then used to define coordinated multi-measurement responses. Specifically, co-elevation events were assessed for the ALT–AST, ALP–TBIL, ALP–GGT/GTP, and BUN–CRE pairs, as well as the ALT–AST–LDH triad, with a co-elevation event recorded only when all constituent measurements met the elevation criteria. Agreement between real-control- and synthetic-control-based co-elevation calls was quantified using recall (fraction of treatments co-elevated under real controls that were also co-elevated under synthetic controls), specificity (fraction of treatments not co-elevated under real controls that remained not co-elevated under synthetic controls), and balanced accuracy (the mean of recall and specificity).

#### Toxicity assessment with synthetic controls

We analyzed GanCtrl’s ability to replicate treatment-effect outcomes by implementing concordance analysis comparing treatment-effect calls derived using real-control references versus synthetic-control references. For each treatment group and measurement, every treatment was standardized against each control source using a control-referenced z-score:


$$z=\frac{x_{\mathrm{treatment}}-\mu_{\mathrm{control}}}{\sigma_{\mathrm{control}}},$$

where $x_{\mathrm{treatment}\;}$denotes the observed value in the treatment group, and $\mu_{\mathrm{control}\;}$and $\sigma_{\mathrm{control}}$ represent the mean and SD of the corresponding control group (real or synthetic).

In the training set, z-scores computed using real controls were first used to assign reference labels: Samples with |z|>2 were labeled positive and those with |z|≤2 were labeled negative. A treatment was then classified as positive if at least one of its samples was positive; otherwise, it was classified as negative. Using synthetic controls for the same samples, we then computed *z*-scores with a per-measurement threshold selected to maximize concordance with the real-referenced training labels. The threshold was chosen from a search range of T∈{1, …,20}. Concordance agreement was defined by synthetic-control-referenced calls against real-referenced calls at the treatment level into true positives (positive under both), true negatives (negative under both), false positives (FP, positive under GanCtrl but negative under real), and false negatives (FN, positive under real but negative under GanCtrl), and computed as:


$$\mathrm{Concordance}\;\mathrm{Agreement}=\frac{TP+TN}{TP+TN+FP+FN}$$

On the test set, real-referenced labels continued to use |z|>2 for positive and |z|≤2 for negative classification, whereas synthetic-control-referenced labels used the training-selected, measurement-specific thresholds T. We then computed treatment-level concordance agreement.

#### VCG approach

To benchmark GanCtrl against current VCG approaches, we adapted previously described VCG construction principles (Lotfi et al. 2026) to the TG-GATEs dataset. The historical control data (HCD) used as the VCG source pool comprised training-set concurrent controls from 73 compounds, along with relevant study metadata, including vehicle and testing facility/laboratory. Detailed VCG inclusion criteria and construction parameters are provided in Table S2.

For each test compound–time group, VCGs were generated by randomly selecting historical control animals from the HCD that had the same sacrifice time, vehicle, and laboratory as that test group. Controls from the same compound were excluded to prevent information leakage. Each VCG was sampled to have the same number of animals as the corresponding real concurrent control group in the test set, and this sampling was repeated 100 times using independent random draws from the eligible HCD pool.

We also generated lab-relaxed VCGs (VCG-LR), in which historical controls were matched on sacrifice time and vehicle but could come from different laboratories. This lab-relaxed, mixed-laboratory setting approximates a real-world scenario in which historical controls may be pooled across testing facilities.

## Results

**Fig. 1.**
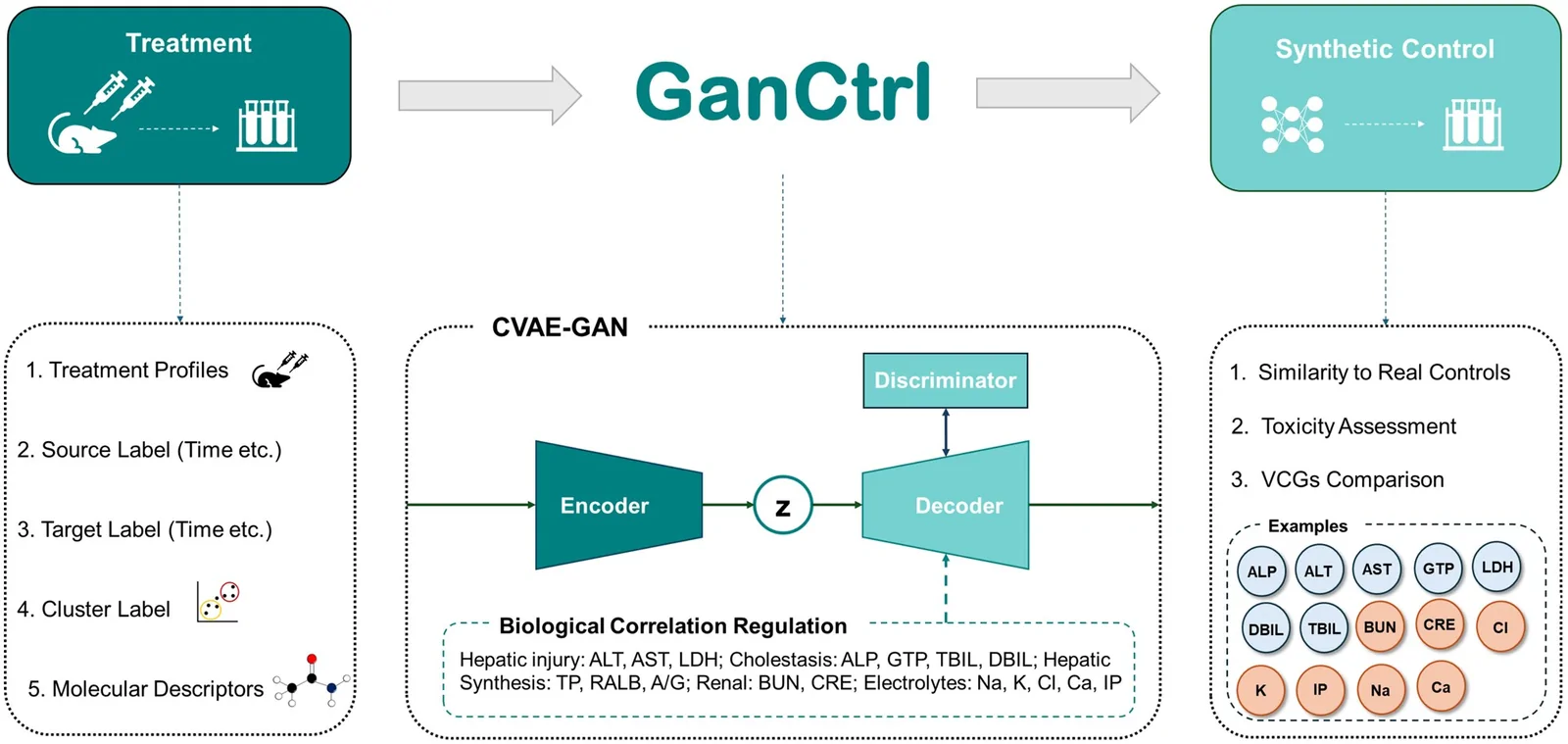
Overview of the GanCtrl workflow. GanCtrl generates synthetic controls data from time-matched treatment clinical pathology data. The model is based on a CVAE-GAN, which integrates a conditional variational autoencoder (CVAE) with a generative adversarial network (GAN). In this architecture, the encoder maps treatment-related information and conditioning variables into a latent representation, *z*. These inputs include values across 38 clinical pathology measurements; source and target context variables, such as time, replicate, and body weight; a cluster label; and Mordred-derived 2D molecular descriptors. The decoder/generator then uses this latent representation to reconstruct or generate synthetic control profiles. A discriminator is used to distinguish generated profiles from real control profiles, encouraging the model to produce realistic and distributionally consistent synthetic control data. To improve biological plausibility, GanCtrl incorporates biological correlation regulation, which constrains generated outputs to preserve known relationships among clinical pathology endpoints. The decoder outputs synthetic control profiles across the 38 clinical pathology endpoints. Model performance was assessed on the test set based on similarity to real animal controls, utility for toxicity assessment, and performance relative to virtual control groups (VCGs).

The dataset was split into the training and test sets by compounds. GanCtrl was implemented as a CVAE–GAN translator (Fig. 1) trained on 10,369 treatment–control pairs from 110 compounds (the training set). The model learned a latent representation of treatment profiles (through VAE) and generated time-matched synthetic controls (through GAN). The model was then evaluated on the test set of 2,566 paired samples from 28 compounds.

GanCtrl performance was assessed around 3 key questions: (i) How closely does GanCtrl reproduce the characteristics of real control animals? (ii) Can synthetic controls be reliably used to support toxicological assessment? (iii) How does GanCtrl differ from VCGs?

### GanCtrl versus real animal controls

**Fig. 2.**
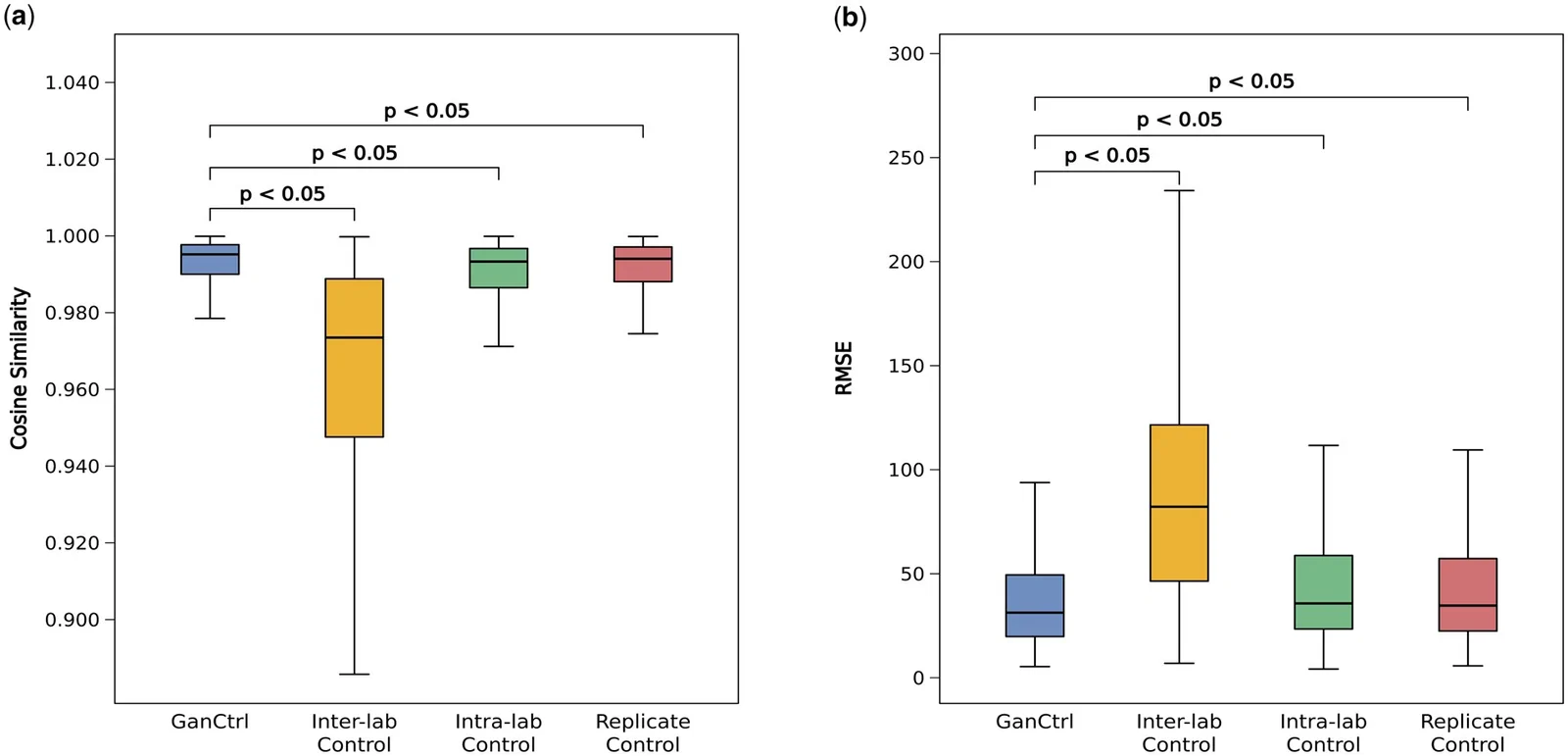
Similarity of GanCtrl-generated synthetic controls to real controls. Similarity between synthetic and real control clinical pathology profiles was evaluated across 38 endpoints using a) cosine similarity and b) root mean squared error (RMSE). Benchmark control groups were included to contextualize model performance: the inter-lab control group compares real control profiles from studies using the same vehicle but conducted in different laboratories; the intra-lab control group compares real control profiles from studies using the same vehicle within the same laboratory; and the replicate control group compares biological replicate controls within the same compound–time group.

We assessed agreement between synthetic and real control data in the test set using cosine similarity and RMSE, benchmarking these metrics against 3 baselines derived from time-matched real controls. These baselines provided contextual reference for expected biological variability: An inter-laboratory baseline (same vehicle, different laboratories), an intra-laboratory baseline (same vehicle and laboratory), and a replicate baseline (biological replicates within the same study). Inter- and intra-laboratory baselines were estimated using the 86 compounds with available vehicle–laboratory metadata, whereas the replicate baseline was computed across all compounds. As depicted in Fig. 2, GanCtrl consistently exceeded both inter- and intra-laboratory baselines, showing higher cosine similarity (0.9920 vs. 0.9638 and 0.9896; *P* < 0.05) and lower RMSE (40.4113 vs. 90.7139 and 46.7519; *P* < 0.05). Relative to the replicate baseline, GanCtrl also achieved higher similarity (0.9920 vs. 0.9905; *P* < 0.05) and lower RMSE (40.4113 vs. 45.2063; *P* < 0.05). These results indicate that GanCtrl generated synthetic control profiles with replicate-level similarity across studies and laboratories, supporting its potential to reduce real controls in animal studies.

**Fig. 3.**
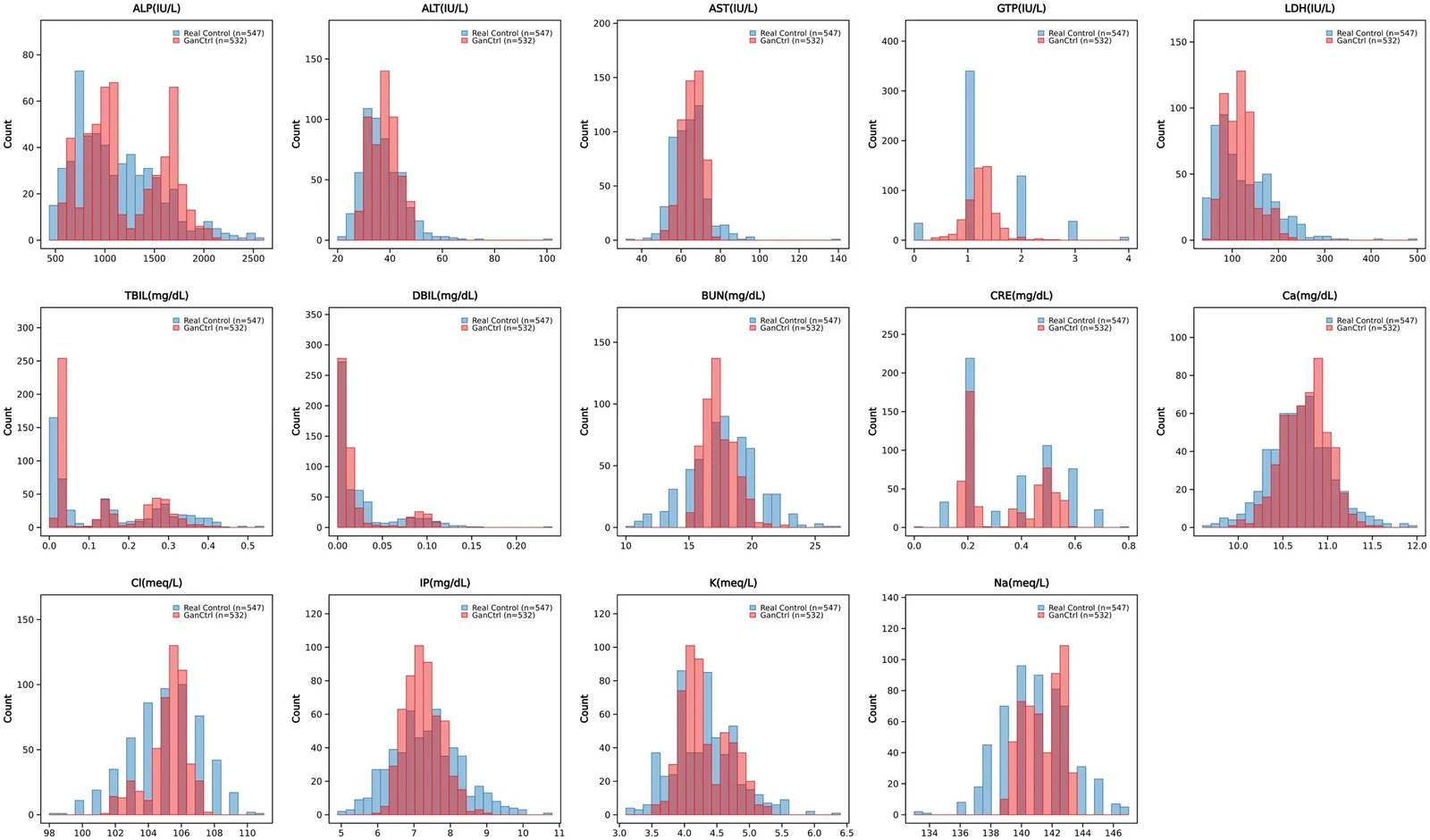
Comparison of clinical pathology endpoint distributions in real and GanCtrl-generated synthetic controls. Distributions of real and synthetic control values were compared across 14 hepatotoxicity- and nephrotoxicity-related clinical pathology endpoints. Overlaid histograms show per-animal values for real concurrent controls and GanCtrl-generated synthetic controls, pooled across all treatment groups. In each panel, the x-axis shows endpoint measurements in their original units, and the y-axis shows animal counts.

We also assessed the distributional similarity of synthetic controls relative to real controls for each measurement. We quantified these differences using the Jensen–Shannon divergence, a symmetric measure of distributional similarity (Lin 1991; Öngün and Temizel 2021). Generally, the synthetic controls showed close alignment with real controls, with modest divergences across all 38 measurements (median ≈ 0.19, where 0 denotes identical distributions). We further illustrated the distributions for the 14 hepatotoxicity- and nephrotoxicity-related measurements in Fig. 3. Together, these results indicate that GanCtrl not only generates control profiles at the sample level but also preserves the magnitude and distribution of control responses across studies.

**Fig. 4.**
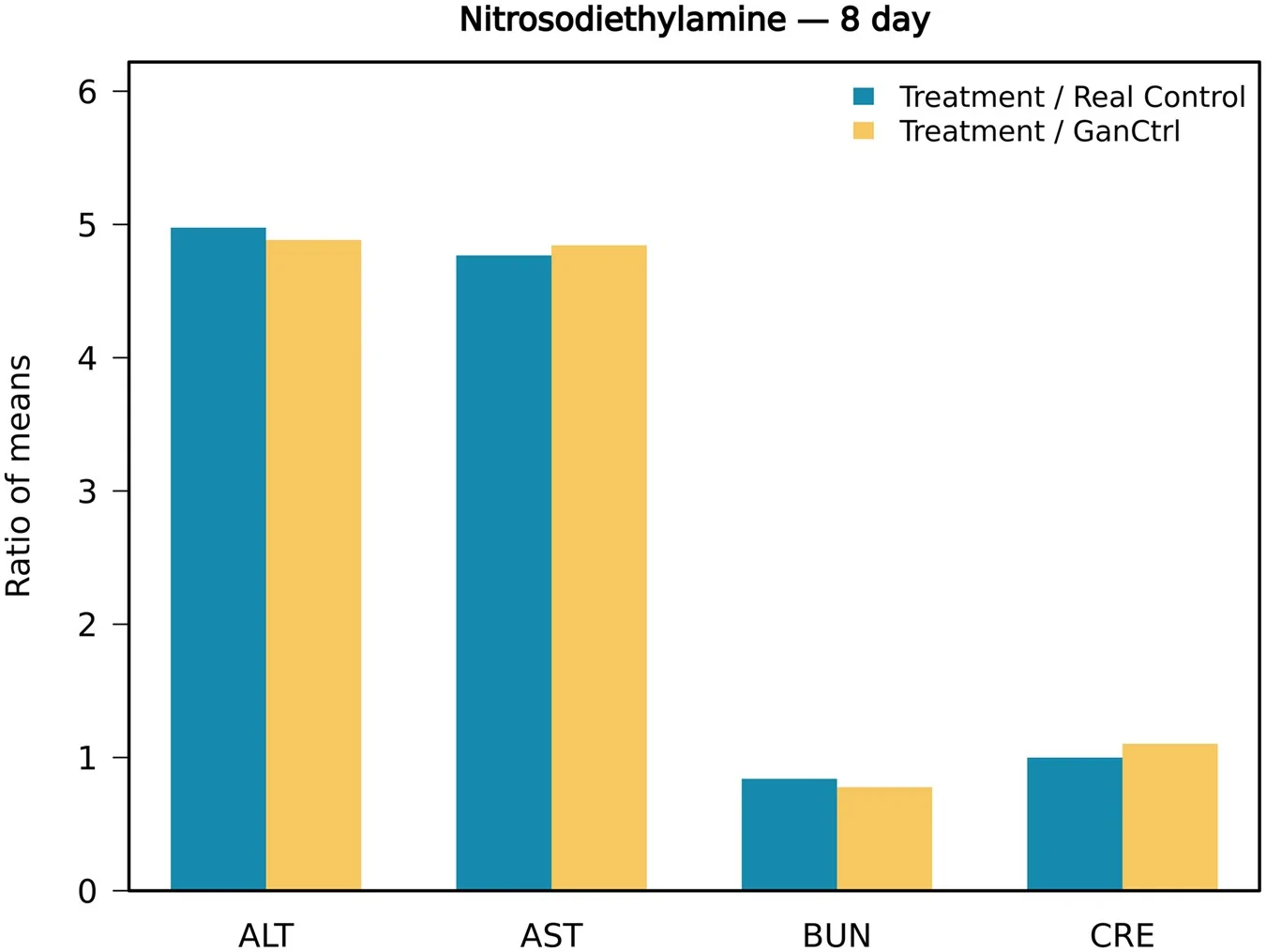
Preservation of coordinated multi-measurement responses with GanCtrl-derived synthetic controls. Treatment-to-control ratios for selected liver- and kidney-injury-related endpoints in a representative 8-day nitrosodiethylamine exposure case. Ratios were calculated using either real controls or GanCtrl-generated synthetic controls, with a ratio of 1 indicating no difference between the treatment and corresponding control group. Bar plots illustrate whether coordinated organ-injury response patterns are maintained when synthetic controls are used in place of real controls.

Prior studies have shown that specific clinical pathology endpoints associated with liver or kidney toxicity exhibit co-occurring expression patterns. We used these established co-expression patterns to assess whether synthetic controls reflected the same biological relationships observed in real controls. As reported (Latief et al. 2016; Hussain et al. 2025), nitrosodiethylamine primarily induces liver rather than kidney injury, characterized by ALT and AST co-elevation; similarly, our generated control data reproduces this ALT–AST pattern while accurately reflecting the absence of BUN and CRE co-elevation observed in the real dataset. As shown in Fig. 4, the expression patterns in both organs were recapitulated with synthetic controls. The complete co-expression analysis results are summarized in Table S3.

### Toxicity assessment with synthetic controls

Toxicology assessments rely on the time-matched control to judge whether a treated sample exhibits a treatment-related deviation that affects the safety margin (Chen et al. 2023). Using the test set, we evaluated concordance between treatment-level effect calls derived from synthetic controls and those obtained using real controls, with overall concordance reaching 0.70 across the 14 endpoints; endpoint-level results are reported in Table S4.

**Fig. 5.**
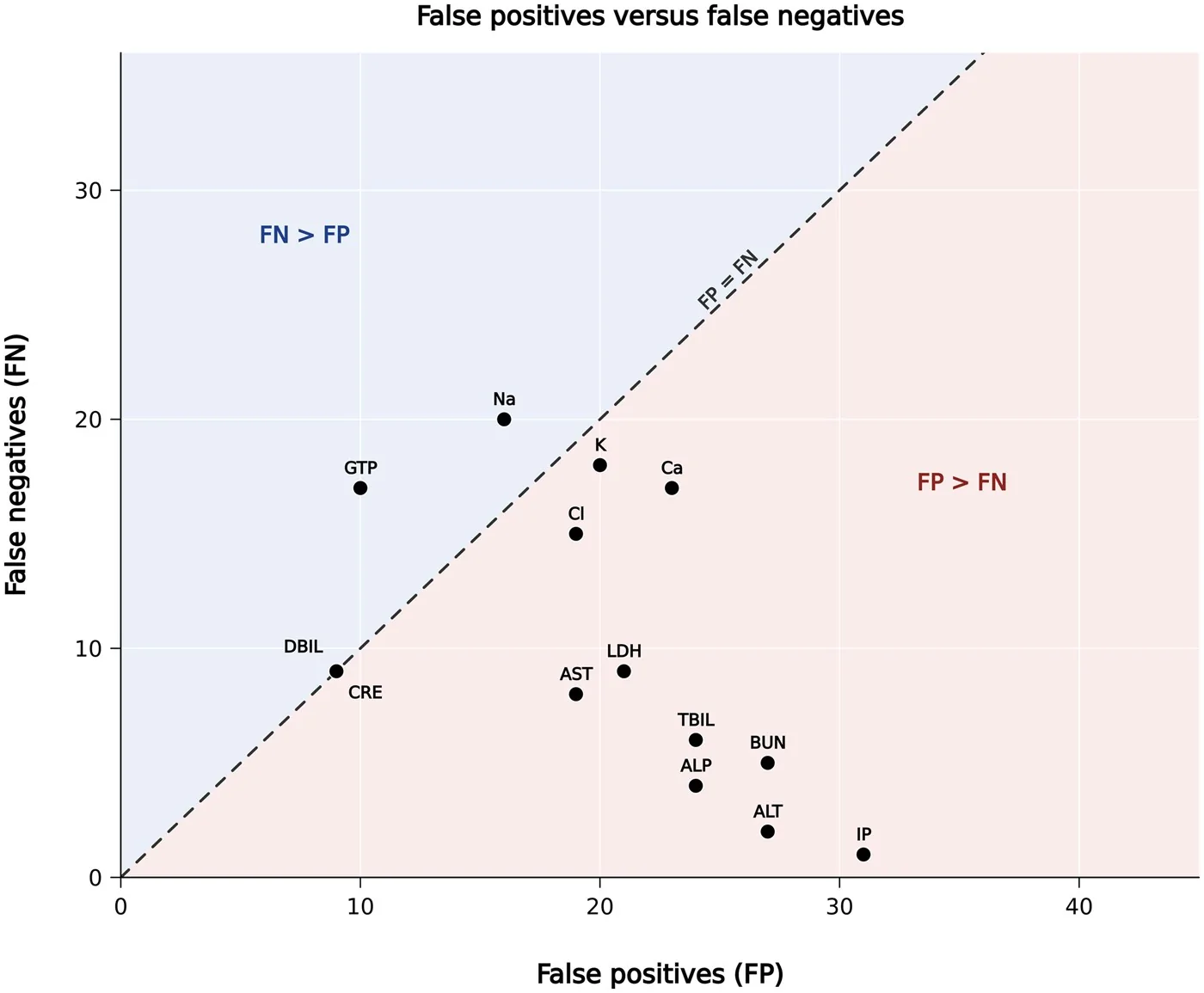
False-positive versus false-negative calls across hepatotoxicity- and nephrotoxicity-associated endpoints. False-positive (FP) and false-negative (FN) treatment-effect calls were summarized for the 14 hepatotoxicity- and nephrotoxicity-related endpoints evaluated in the concordance analysis. Each point represents 1 endpoint, with FP counts on the *x* axis and FN counts on the *y* axis. The dashed diagonal line denotes equal numbers of FP and FN calls. Points above the line indicate endpoints with relatively more FN than FP calls, whereas points below the line indicate endpoints with relatively more FP than FN calls. The shaded regions highlight these 2 endpoint-level error-profile patterns.

We further analyzed FP and FN treatment-effect calls across the 14 endpoints to better characterize GanCtrl’s error profile. As shown in Fig. 5, most endpoints fell below the diagonal line, indicating that FP calls were more frequent than FN calls for the majority of endpoints. Because FN calls may lead to missed toxicity signals, we further evaluated endpoint-level sensitivity to assess GanCtrl’s ability to detect treatment-related effects. GanCtrl showed high sensitivity for several clinically relevant endpoints, including IP (98.5%), ALT (97.3%), ALP (93.7%), BUN (92.1%), and TBIL (90.5%), although lower sensitivity was observed for GTP (45.2%) and Na (60.0%). Detailed sensitivity, specificity, and balanced accuracy results are reported in Table S4.

### GanCtrl versus VCGs

**Fig. 6.**
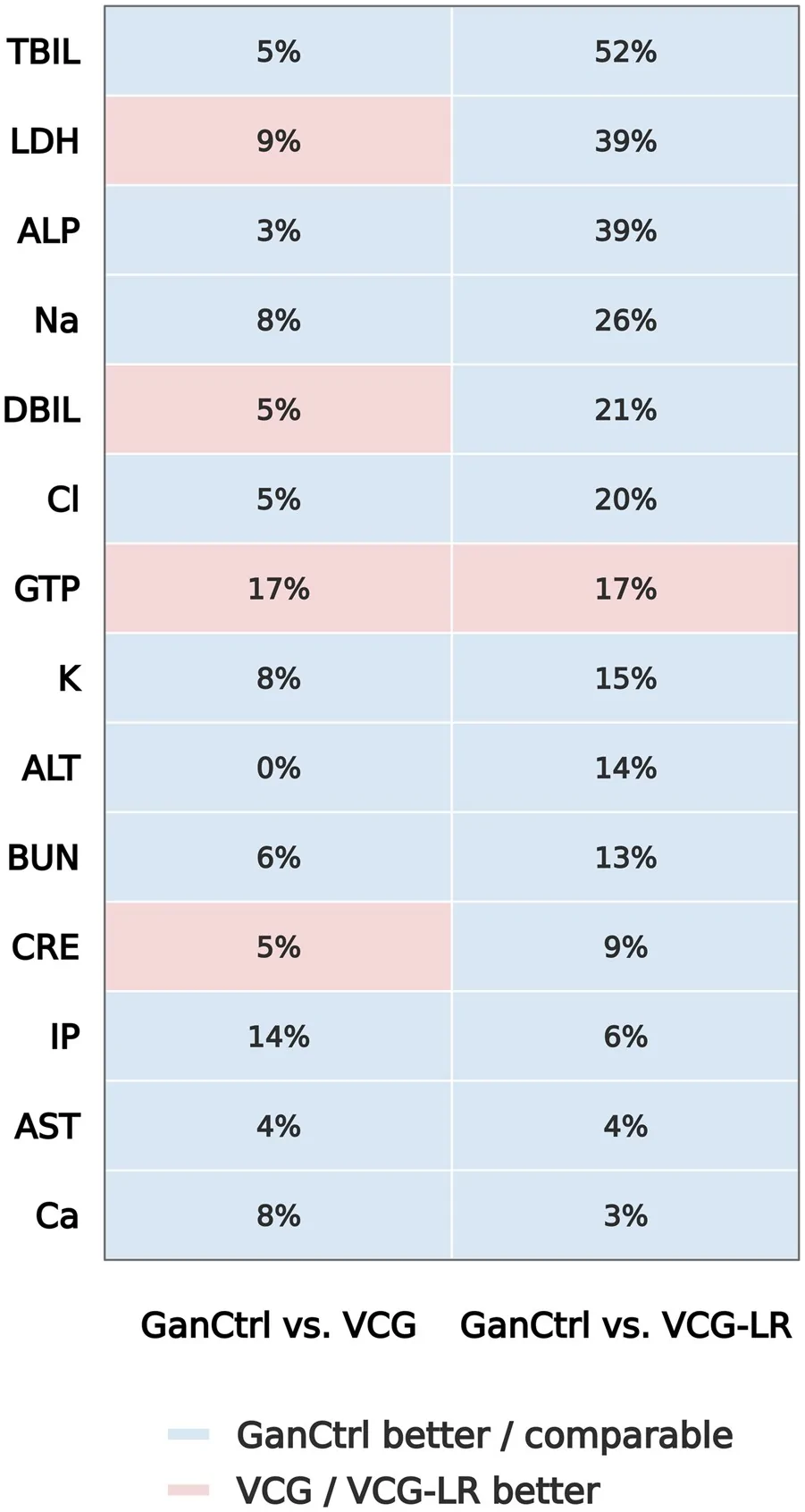
Relative concordance performance of GanCtrl compared with VCGs. For each clinical pathology measurement, concordance analysis was used to compare treatment-effect calls based on GanCtrl-derived synthetic controls against those obtained using real concurrent controls. Benchmarking was performed against VCG derived from a single laboratory and laboratory-relaxed VCG-LR using the data from different laboratories. For each endpoint, the cell value represents the percent relative difference in concordance between GanCtrl and each VCG baseline. Blue cells indicate endpoints where GanCtrl achieved better or comparable concordance, whereas red cells indicate endpoints where the VCG or VCG-LR baseline achieved better concordance. Results are shown across 14 clinically relevant liver- and kidney-related endpoints.

The concordance results from GanCtrl were also benchmarked against current VCG approaches, including a VCG built using only data from a single laboratory and a VCG-LR built by combining data from different laboratories. Figure 6 shows the endpoint-level percent relative difference in concordance for GanCtrl compared with the VCG and VCG-LR baselines across 14 clinically relevant clinical pathology endpoints used in hepatotoxicity and nephrotoxicity assessment. Overall, GanCtrl achieved an average concordance of 0.70, which was comparable to the VCG baseline (0.69) and higher than the VCG-LR baseline (0.61). Consistent with this pattern, the difference between GanCtrl and VCG was not statistically significant (*P *= 0.664), indicating that GanCtrl performed similarly to the standard VCG baseline. In contrast, GanCtrl significantly outperformed VCG-LR (*P *= 0.003), demonstrating stronger concordance than the results relied on VCG-LR baseline.

## Discussion

The 3Rs-driven paradigm has accelerated the adoption of NAMs (Kaplan et al. 2024; Carratt et al. 2025; Stoykova 2025; von Keutz 2025), including coordinated, multi-institutional initiatives that leverage large historical control datasets to reduce reliance on concurrent control animals (Houser and Mangipudy 2019; Steger-Hartmann et al. 2025). Although historical-data-based VCGs represent an important advance, their performance can be affected by “batch effects” such as dataset heterogeneity, differences in study design, animal husbandry, laboratory procedures, and biological drift over time (Steger-Hartmann et al. 2020; Grevot et al. 2023; Golden et al. 2024; Gurjanov et al. 2024; Li et al. 2024a; Adedeji and Naor 2025). To address these challenges, we proposed GanCtrl, a GenAI-based framework that infers control data directly from time-matched treatment data, generating synthetic controls analogous to concurrent controls in real experiments. This design minimizes the batch effects by focusing on individual experiment-specific baselines.

The central assumption underlying GanCtrl is that time-matched control data can be inferred from treatment data because, within an adaptive biological range, treated samples retain information about their underlying control-like physiological state despite treatment-induced perturbations. This assumption is grounded in the biological principle of homeostasis, whereby mammalian systems continuously regulate physiological processes to maintain internal equilibrium and, following perturbations that remain within adaptive limits, tend to return toward baseline conditions. This assumption is particularly relevant to preclinical toxicology studies, where dose selection is intended to elicit measurable biological responses while avoiding excessive systemic toxicity. In the TG-GATEs in vivo studies, the highest dose was selected to induce the minimum toxic effect, rather than a lethally toxic response. Consequently, many treatment-induced changes in this dataset are expected to represent adaptive or partially reversible responses, preserving sufficient information for reconstructing the corresponding control state rather than reflecting complete disruption of normal physiology.

VCG approaches that rely on historical data are based on the premise that toxicological findings from future treatment experiments can be interpreted using virtual controls constructed from prior studies. In other words, the treated and control data are generated under different experimental settings, even if both studies are conducted in the same laboratory. The time gap between experiments may introduce changes in experimental conditions, personnel, instrumentation, environmental factors, or other sources of variability, making this approach analogous to use of data generated by different laboratories. For that, we compared GanCtrl with VCG in 2 settings: single-laboratory data and mixed-laboratories data. Although both approaches performed comparably when single-laboratory data were used, GanCtrl significantly outperformed VCG when built on mixed-laboratories data, demonstrating its utility in real-world scenarios.

By examining GanCtrl results across all endpoints, we found that most endpoints produced more FP than FN predictions, indicating that GanCtrl adopts a relatively conservative approach to identifying abnormal effects. This characteristic is desirable for a screening tool because FP findings can be resolved through subsequent experimental testing, whereas FN predictions may allow potentially toxic compounds to advance without further evaluation, increasing the risk of missed safety signals and potential harm. We also observed that some endpoints exhibited substantial inter-animal variability, such as GTP, which showed a relatively higher FN rate. This finding suggests that the intrinsic variability of an endpoint can limit model performance, making subtle treatment-related changes more difficult to distinguish from normal biological variation.

To determine whether GanCtrl preserves biologically meaningful control characteristics, we evaluated its performance from 3 complementary perspectives. First, we assessed sample-level agreement between synthetic and real controls and compared GanCtrl against expected biological variability baselines. GanCtrl showed stronger agreement than inter- and intra-laboratory baselines and performed comparably to biological replicates. These findings indicate that GanCtrl-generated controls approximate real concurrent controls within the expected range of biological replicate variability.

Second, we assessed endpoint-level distributional similarity using Jensen–Shannon divergence. Synthetic controls showed close alignment with real controls across all 38 clinical pathology measurements, with only modest divergence overall. This pattern was further illustrated for the 14 hepatotoxicity- and nephrotoxicity-related endpoints, supporting that GanCtrl preserves not only sample-level profiles but also the magnitude and distribution of control responses across studies.

Third, we examined whether GanCtrl maintained established biological relationships among clinical pathology endpoints. Many endpoints exhibit co-occurring changes because they reflect common underlying physiological or pathological processes. For example, during hepatocellular injury, ALT and AST are typically elevated together as damaged hepatocytes release both enzymes into the circulation (Kobayashi et al. 2020; Thakur et al. 2024), whereas BUN and CRE commonly increase together during renal dysfunction as glomerular filtration declines (Edelstein 2017; Abd El-Rhman et al. 2020). GanCtrl successfully preserved these expected patterns. Likewise, other well-established endpoint relationships—including ALP–GGT for cholestatic injury (Atshan and Zalzala 2024), ALP–TBIL for impaired bile metabolism (Yan et al. 2022), and the coordinated elevation of ALT, AST, and LDH during severe acute hepatic injury (Muratsubaki and Yamaki 2011)—were maintained. These findings demonstrate that GanCtrl preserves not only individual endpoint distributions and treatment effects but also the underlying biological correlation structure, likely owing to its biologically informed attention mask and correlation-structure loss.

Applicability domain is an important consideration in predictive toxicology, particularly for Quantitative Structure–Activity Relationship models and other structure-based AI models, where chemical structure serves as the primary or sole model input. GanCtrl differs fundamentally from these approaches because it integrates multiple sources of information, including treatment-related clinical pathology measurements (e.g. ALT and AST), in addition to compound identity. Consequently, its applicability domain should be considered in a broader biological context rather than being defined solely by chemical structural similarity. This concept is supported by our previous work on AnimalGAN (Chen et al. 2023), a GenAI model developed using the Open TG-GATEs dataset. In that study, we evaluated model generalizability using 3 train–test splitting strategies based on chemical structure, therapeutic class, and drug approval year. Model performance remained largely consistent across all 3 strategies, indicating that chemical structural similarity was not the primary determinant of predictive performance. To further assess the applicability domain of GanCtrl, we evaluated compounds whose primary mechanisms of toxicity were underrepresented in the training data. These included lipid/PPAR-related compounds (WY-14643, clofibrate, fenofibrate, and nicotinic acid), as well as penicillamine and allopurinol, representing less common immunomodulatory and purine/uric acid metabolism mechanisms, respectively. GanCtrl achieved performance comparable to that observed for compounds belonging to well-represented mechanistic classes. These results suggest that the model generalizes beyond the dominant mechanisms present in the training set and that its applicability domain is driven by learned biological response patterns rather than chemical similarity alone.

It is worth noting that this study is intended as a proof-of-concept framework rather than an immediately deployable tool for external toxicology datasets, and a few limitations should be kept in mind. The current analysis draws on Open TG-GATEs, a well-curated but inherently bounded dataset, and GanCtrl performance would benefit from further evaluation across a wider range of study designs, laboratories, and analytical platforms. Additionally, the model was assessed using highest-dose groups, which offer a useful but narrow benchmark for inferring study-aligned synthetic controls from treated profiles; how well the approach performs across lower-dose response settings, where treatment effects tend to be more subtle and closer to baseline variability, remains an open question. The biological scope is also limited to clinical pathology samples from male Sprague–Dawley rats, and whether findings extend to female animals, other strains, or additional species will require further investigation. Although GanCtrl’s time-matched treatment–control pairing is designed to help reduce study-associated baseline variability, they may not fully separate biological variability from laboratory- or platform-driven technical sources. Lastly, GanCtrl has not been tested on compounds or experimental conditions that induce irreversible tissue damage, severe toxicity, excessive dosing, or prolonged biological perturbations that overwhelm homeostatic mechanisms.

In summary, this proof-of-concept pilot study demonstrated that AI-based generative models can be used to construct dynamic, study-specific synthetic controls from treatment samples for toxicity studies. To our knowledge, GanCtrl is the first AI-assisted synthetic control modeling approach to apply a CVAE–GAN architecture that explicitly models biological coherence, animal-level variability, and study effects observed in real experiments. By conditioning on the treated cohort and experimental context, GanCtrl provides a biologically contextualized approach for constructing VCGs. Looking ahead, future work will explore GanCtrl’s performance using independent large datasets to better understand how broadly the framework may generalize across biological, technical, and experimental contexts. Most importantly, the model evaluation should define a clear context of use, include external validation, and characterize model uncertainty and potential failure modes. With continued validation, AI-derived synthetic controls may support more reliable toxicity assessment while progressively reducing dependence on concurrent control animals, consistent with the 3Rs principles and ongoing efforts to modernize preclinical safety testing.

## Supplementary Material

kfag099_Supplementary_Data

## Data Availability

The datasets used to develop and evaluate GanCtrl are curated from the Open TG-GATEs database available for download at https://dbarchive.biosciencedbc.jp/en/open-tggates/download.html. The training and test input files have been deposited at Zenodo under https://doi.org/10.5281/zenodo.17883691.

## Code availability

GanCtrl was developed using tensorflow-GPU version 2.4.1 with open-source Python (version 3.11.7). The visualization was done in R (version 4.4.1). The source code is available at CHANDMX20/GanCtrl.
