# Supplementary material for: GanCtrl: a generative AI approach to derive study-aligned synthetic controls for reducing concurrent control animal use
Source: Toxicol Sci. 2026 Aug 6;209(8):kfag099. doi: 10.1093/toxsci/kfag099 (PMC13505625; doi:10.1093/toxsci/kfag099)
Supplement: Supplementary file 1 [file kfag099_supplementary_data.docx]

***GanCtrl:* A Generative AI Approach to Derive Study-Aligned Synthetic Controls for Reducing Concurrent Control Animal Use**

**Mansi Chandra^1,2^, Xi Chen^1^, Ting Li^1,^ *, and Weida Tong^1^**

**^1^ National Center for Toxicological Research, Food and Drug Administration, Jefferson, Arkansas 72079, USA.**

**^2^ University of Arkansas at Little Rock and University of Arkansas for Medical Sciences Joint Bioinformatics Program, Little Rock, Arkansas 72204, USA**

*** Corresponding author: Ting Li (**[**Ting.Li@fda.hhs.gov**](mailto:Ting.Li@fda.hhs.gov)**)**

**Supplementary Table S1:** *Liver and kidney-specific measurements panels used*

| *Liver panel* | |
| --- | --- |
| ALP (IU/L) | Alkaline phosphatase |
| ALT (IU/L) | Alanine aminotransferase |
| AST (IU/L) | Aspartate aminotransferase |
| GTP (IU/L) | γ-glutamyltranspeptidase |
| LDH (IU/L) | Lactate dehydrogenase |
| DBIL (mg/dL) | Direct bilirubin |
| TBIL (mg/dL) | Total bilirubin |
| *Kidney panel* | |
| BUN (mg/dL) | Blood urea nitrogen |
| CRE (mg/dL) | Creatinine |
| Cl (meq/L) | Chlorine |
| Ca (mg/dL) | Calcium |
| K (meq/L) | Potassium |
| IP (mg/dL) | Inorganic phosphorus |
| Na (meq/L) | Sodium |

**Supplementary Table S2:** *Selection Criteria for TG-GATEs Historical Control Data Used to Construct VCG Baselines.*

| Species | Rat |
| --- | --- |
| Strain | Sprague-Dawley |
| Sex | Male |
| Age | 6 weeks |
| Body weight range | 165–242.7 g |
| Dosing durations | 3, 7, 14, and 28 days |
| Testing facility/laboratory | A, B, C, and D |
| Number of studies | 73 |
| Standard VCG | Same sacrifice time, vehicle, and laboratory |
| Lab-relaxed VCG | Same sacrifice time and vehicle, different laboratories allowed |
| Sampling strategy | Random sampling repeated 100 times, with VCG size matched to the corresponding real concurrent control group |

**Supplementary Table S3:** *Coordinated multi-measurement responses (co-elevation) across all test set compound-time groups*

| Measurement Combination | Real co-elevated groups | TP | TN | FP | FN | Recall | Specificity | Balanced Accuracy |
| --- | --- | --- | --- | --- | --- | --- | --- | --- |
| ALT-AST | 3 | 3 | 102 | 1 | 0 | 1.000 | 0.990 | 0.995 |
| ALP-TBIL | 2 | 2 | 103 | 1 | 0 | 1.000 | 0.990 | 0.995 |
| ALP-GTP | 1 | 1 | 105 | 0 | 0 | 1.000 | 1.000 | 1.000 |
| BUN-CRE | 1 | 1 | 102 | 3 | 0 | 1.000 | 0.971 | 0.986 |
| ALT-AST-LDH | 1 | 1 | 105 | 0 | 0 | 1.000 | 1.000 | 1.000 |

**Supplementary Table S4:** *Classification errors and performance metrics by endpoint across all test set compounds*

| Endpoint | TP | TN | FP | FN | Accuracy | Balanced Accuracy | Sensitivity | Specificity |
| --- | --- | --- | --- | --- | --- | --- | --- | --- |
| ALP | 59 | 20 | 24 | 4 | 73.8 | 69.6 | 93.7 | 45.5 |
| ALT | 72 | 6 | 27 | 2 | 72.9 | 57.7 | 97.3 | 18.2 |
| AST | 67 | 13 | 19 | 8 | 74.8 | 65 | 89.3 | 40.6 |
| GTP | 14 | 39 | 10 | 17 | 66.2 | 62.4 | 45.2 | 79.6 |
| LDH | 64 | 13 | 21 | 9 | 72 | 63 | 87.7 | 38.2 |
| DBIL | 27 | 24 | 9 | 9 | 73.9 | 73.9 | 75 | 72.7 |
| TBIL | 57 | 15 | 24 | 6 | 70.6 | 64.5 | 90.5 | 38.5 |
| BUN | 58 | 17 | 27 | 5 | 70.1 | 65.3 | 92.1 | 38.6 |
| CRE | 16 | 35 | 9 | 9 | 73.9 | 71.8 | 64 | 79.5 |
| Cl | 35 | 37 | 19 | 15 | 67.9 | 68 | 70 | 66.1 |
| Ca | 39 | 28 | 23 | 17 | 62.6 | 62.3 | 69.6 | 54.9 |
| K | 38 | 31 | 20 | 18 | 64.5 | 64.3 | 67.9 | 60.8 |
| IP | 66 | 9 | 31 | 1 | 70.1 | 60.5 | 98.5 | 22.5 |
| Na | 30 | 41 | 16 | 20 | 66.4 | 66 | 60 | 71.9 |

**Supplementary Methods**

**GanCtrl Blueprint Formulation**

**A) Masked Attention-aware Mixing**

The decoder first mixes treatment inputs with a masked, row-wise softmax attention:

$$S={softmax}_{row}\left( A ⨀ M+ \left( 1-M \right)\cdot\left( -\infty\right) \right), \tilde{x}=Sx^{treatment}$$

where $A\in\mathbb{R}^{A\times A}$ are learned logits (via $\mathcal{l}_{s}, \mathcal{l}_{t}$ context), $M$ is the fixed biology mask (hepatic/renal/electrolytes groups). This yields $\tilde{x}$ that diffuses information only within biologically permitted neighborhoods.

**B) Biologically Informed Decoder Output**

From the shared backbone, the decoder aggregates a base map and cluster-specialized predictions via one-hot cluster vector. Mathematically, this can be expressed as:

$$y=W_{0}\left( h \right)+ \sum_{k=1}^{K} c_{k}f_{k}\left( h \right),$$

where $h$ denotes the shared base representation, $f_{k}\left( . \right)$ is the $k^{th}$ sub-decoder, $c_{k}$ is the one-hot batch gating weight, and $W_{0}$is the shared base mapping. The final heads are

$$\mu= \sigma\left( W_{\mu}y \right), \log\sigma^{2}= W_{v}h_{v}$$

with $\sigma\left( \cdot\right)$ the logistic sigmoid: $\mu\in\left( 0,1 \right)^{A}, \log\sigma^{2}\in$ $\mathbb{R}^{A}$ (heteroscedastic output).

**Mathematical Formulation of the GanCtrl Training Objective and Loss Functions**

The *GanCtrl* framework was optimized using a composite loss function that integrates reconstruction fidelity, latent regularization, adversarial alignment, and biologically guided constraints. The total training objective is expressed as:

$$\mathcal{L}_{total}= \omega_{NLL}\mathcal{L}_{NLL}+ + \beta_{KL}\mathcal{L}_{KL}+ \omega_{adv}\mathcal{L}_{adv}+ \omega_{range}(\mathcal{L}_{range, \mu}+ \mathcal{L}_{range, x})+ {\lambda_{bio-corr}\mathcal{L}}_{bio-corr}+ {\lambda_{mean-match}\mathcal{L}}_{mean-match}$$

**A) Variation Reconstruction Loss**

The core reconstruction objective follows the Gaussian negative log-likelihood (NLL):

$$\mathcal{L}_{NLL}= \frac{1}{2}\sum_{i} \left[ \log\sigma_{i}^{2}+\frac{{(x_{i}-\mu_{i})}^{2}}{\sigma_{i}^{2}} \right],$$

where $x_{i}$ denotes the real control feature value, and $\mu_{i}$ and $\sigma_{i}^{2}$ represent the predicted mean and variance for each measurement.

The KL divergence term regularizes the latent space by aligning the learned posterior distribution with an isotropic Gaussian prior:

$$\mathcal{L}_{KL}= -\frac{1}{2}\sum_{j} \left[ 1+\log{(\sigma}_{z,j}^{2})-\mu_{z,j}^{2}-\sigma_{z,j}^{2} \right],$$

where, $\mu_{z,j}$ and $\sigma_{z,j}^{2}$denote the encoder-derived mean and variance of latent dimension $j$.

**B) Adversarial Loss**

To improve distributional realism, an adversarial discriminator is trained alongside the decoder. The decoder’s adversarial objective is defined as:

$$\mathcal{L}_{adv}= -\mathbb{E}_{x_{real}}\left[ \log D\left( x_{real} \right) \right]- \mathbb{E}_{x_{fake}}\left[ \log\left( 1-D(x_{fake}) \right) \right]$$

where $D(.)$ denotes the discriminator output. Label smoothing (0.1) was applied to improve stability.

**C)** **Physiological Range Constraint**

To prevent physiologically implausible outputs, a range-based loss was applied to total bilirubin (TBIL):

$$\mathcal{L}_{range}\mathbb{= E}\left[ \max_{} \left( 0, L- x_{TBIL} \right)+ \max_{} \left( 0, x_{TBIL}- H \right) \right],$$

where $L$ and $H$ are the empirically observed lower and upper TBIL limits from real control distributions.

**D)** **Biological Correlation Preservation Loss**

To preserve inter-feature relationships that reflect physiological co-regulation, *GanCtrl* employs a biologically informed correlation loss:

$$\mathcal{L}_{bio-corr}= \sum_{k\in\varepsilon} \omega_{k} \sum_{(i, j)\in P_{k}} \left( r_{ij}^{gen}-r_{ij}^{real} \right)^{2},$$

where $\varepsilon$ is the set of expert panels (e.g., hepatic injury, renal, electrolyte), $P_{k}$ is the set of feature pairs within expert $k$, and $r_{ij}$ is the Pearson correlation between features $i$ and $j$.

**E)** **Mean-Matching Regularization Loss**

A mild alignment constraint ensures that the overall mean of synthetic control profiles remains centered on the real control distribution:

$$\mathcal{L}_{mean-match}= \left\| \mathbb{E}\left[ x_{gen} \right]\mathbb{-E}\left[ x_{real} \right] \right\|_{2}^{2}.$$

**Model Selection Score**

At each training step $t$, we computed a dimensionless composite score by z-normalizing organ-level metrics and combining them with fixed weights:

$$\boldsymbol{C}_{\boldsymbol{t}}\boldsymbol{=}\boldsymbol{\omega}_{\boldsymbol{MM}}\boldsymbol{Z}\left( \boldsymbol{MM}_{\boldsymbol{t}} \right)\boldsymbol{+}\boldsymbol{\omega}_{\boldsymbol{BIO}}\boldsymbol{Z}\left( \boldsymbol{BIO}_{\boldsymbol{t}} \right)\boldsymbol{+}\boldsymbol{\omega}_{\boldsymbol{NLL}}\boldsymbol{Z}\left( \boldsymbol{NLL}_{\boldsymbol{t}} \right)\boldsymbol{+}\boldsymbol{1}_{\boldsymbol{liver}}\boldsymbol{\omega}_{\boldsymbol{TBIL}}\boldsymbol{Z(}\boldsymbol{TBIL}_{\boldsymbol{t}}\boldsymbol{)}$$

Here, ${MM}_{t}$ is the mean-match loss (mean absolute error between synthetic and real controls), ${BIO}_{t}$is the biological correlation loss (upper-triangular mean-squared error between synthetic and real control correlation matrices), ${NLL}_{t}$is a proxy for the training negative log-likelihood, and ${TBIL}_{t}$(liver only, when informative) penalizes bilirubin-range violations. Each component was z-scored across training steps to ensure comparability, and $\omega_{*}$ terms denote the relative weights assigned to each component.
